# Experimental Study and Discrete Analysis of Compressive Properties of Glass Fiber-Reinforced Polymer (GFRP) Bars

**DOI:** 10.3390/polym15122651

**Published:** 2023-06-12

**Authors:** Zhilin Zhou, Long Meng, Feng Zeng, Shuai Guan, Jiahui Sun, T. Tafsirojjaman

**Affiliations:** 1 Zhuhai Xianghai Bridge Co., Ltd., Pingdong Sixth Road 8, Zhuhai 519000, China; zhoulin8785@163.com; 2 Guangxi Xingang Communications Investment Group Co, Ltd., Chenguang Road 100, Qinzhou 535008, China; ml13657800229@163.com; 3 Guangzhou Highway Engineering Group Co., Ltd., Shuimengerheng Road 2, Guangzhou 510170, China; zengfengok@126.com; 4 The Key Laboratory of Urban Security and Disaster Engineering of Ministry of Education, Beijing University of Technology, Pingleyuan Road 100, Beijing 100124, China; guuwalgiya1906@gmail.com (S.G.); sunjiahui@emails.bjut.edu.cn (J.S.); 5 School of Architecture and Civil Engineering, The University of Adelaide, Adelaide 5005, Australia

**Keywords:** GFRP bars, compression test, DIC, Weibull distribution, gamma distribution

## Abstract

Glass fiber-reinforced polymer (GFRP) has superior characteristics over traditional steel, such as lightweight, high strength, corrosion resistance and high durability. GFRP bars can be a useful alternative to steel bars in structures, specifically those in highly corrosive environments, as well as structures subjected to high compressive pressure such as bridge foundations. Digital image correlation (DIC) technology is used to analyze the strain evolution of GFRP bars under compression. It can be seen from using DIC technology that the surface strain of GFRP reinforcement is uniformly distributed and increases approximately linearly, and brittle splitting failure of GFRP bars happens due to locally occurring high strain at the failure stage. Moreover, there are limited studies on the use of distribution functions to describe the compressive strength and elastic modulus of GFRP. In this paper, Weibull distribution and gamma distribution are used to fit the compressive strength and compressive elastic modulus of GFRP bars. The average compressive strength is 667.05 MPa and follows Weibull distribution. Moreover, the average compressive elastic modulus is 47.51 GPa and follows gamma distribution. In order to verify that GFRP bars still have certain strength under compressive conditions, this paper provides a parameter reference for their large-scale application.

## 1. Introduction

Reinforced concrete (RC) structures are severely affected by corrosive environments, leading to the corrosion of steel bars and reducing the strength of components. Compared to iron and steel materials, fiber-reinforced polymer (FRP) has the advantages of being lightweight [1], high strength [2,3], insulation [4,5] and high corrosion resistance [6,7,8] which dramatically increases the use of FRP bars including GFRP bar in civil structures, specifically in adverse and corrosive environment to maintain high safety and durability [9]. As an anisotropic material, the strength of GFRP is affected by many factors, such as fiber content, matrix type and manufacturing process. In order to ensure the safe and efficient use of GFRP, it is necessary to demonstrate its material properties through experiments.

Research on the application of GFRP bars in RC structures began in the 1960s. At that time, the rebars used in highway bridges had serious corrosion problems due to the extensive use of deicing salt [10]. GFRP bars can be used in the construction of bridge foundations, tie beams, columns and other important elements [11] that are not easy to maintain and difficult to replace in the marine environment. These structures are built in the corrosive environment of a marine atmosphere with high temperature, high humidity, strong monsoons, high salinity of seawater and high chlorine content, which may lead to the accelerated corrosion of traditional steel structures and RC structures [12,13]. In this regard, researchers have carried out experimental studies on the properties of GFRP bars, including the mechanical properties of tension, compression and bending, as well as the influence of creep, corrosion and extreme temperature on GFRP bars under long-term service conditions.

In terms of the study of basic mechanical properties, Benmokrane et al. [14] tested the tensile properties of four different types of FRP bar using a bond anchorage scheme with steel anchors at both ends and high-performance resins, to avoid stress concentration leading to the damage of FRP specimens by the testing machine fixture. All the tested specimens showed damage at the free end, which proves that this anchorage form can be used to determine the ultimate tensile strength of GRFP bars. Several studies [15,16,17] conducted experiments on short-column samples or thin-wall structures made of composite materials such as CFRP, and compressed real composite structures until they failed. The test parameters were measured to determine the post-buckling equilibrium path under the whole loading condition, and acoustic emission technology was used to evaluate the initial stage of composite damage. The research method adopted makes it possible to study the nonlinear stability and bearing capacity of compression structures. Researchers [18,19] conducted tensile and compression tests on GFRP bars with smooth lower surfaces of different diameters and found that the compressive strength of GFRP bars was only 50% to 60% of the tensile strength and the compressive elastic modulus of GFRP bars was 80% of the tensile modulus. Kobayashi and Fujisaki [20] tested the ultimate strength of CFRP, GFRP and AFRP bars under monotonic tensile and compressive loads, and then, studied the mechanical properties of the three bars under cyclic tensile and compressive loads, as well. The compressive strengths of AFRP and GFRP bars under cyclic loading were 20 to 50 percent of those under monotone loading, while the mechanical properties of CFRP bars were almost unchanged under cyclic loading. Yu [21] compared the effects of 55%, 60% and 65% fiber content on the compressive strength of GFRP bars with diameters of 8, 10 and 12 mm. It was proven that the higher fiber content corresponded to higher compressive strength, while the diameter had little effect on the compressive strength and elastic modulus. Liu et al. [22] proposed a new pultrusion–winding–pultrusion method to improve the compressive ability of GFRP bars. The compression test proved that the ultimate load of the GFRP bars with a hoop winding layer was increased compared to the GFRP bars without a hoop winding layer. Due to the low compressive strength and ductility of FRP bars, some specifications [23,24] also choose to ignore the influence of FRP bars’ strength when calculating the compressive capacity of RC specimens.

In addition, the behavior of GFRP bars when subjected to elevated temperature also needs to be considered. Mazzuca et al. [25] experimentally and analytically investigated the effect of high temperature on the mechanical properties of GFRP laminates prepared using the vacuum infusion method. The results obtained confirmed that the shear and compression strength of GFRP laminates decreased by about 90% at 200 °C, while the tensile properties (fiber-based) were much less affected, with decreases of about 40%. Wang et al. [26] found that a geopolymer concrete cover can effectively slow down and mitigate degradation of the tensile strength and elastic modulus of GFRP bars after high-temperature exposure (400–600 °C), but this effect weakens following a rise in temperature and time. 

In summary, much research has been conducted on the mechanical properties of FRP bars, especially their tensile properties, but research on the compressive properties of FRP bars, especially their dispersion, is still lacking. In addition, in order to strengthen the bond between FRP bars and concrete, ribs or sticky sand are usually added to the surface of the bars, which makes it difficult to accurately measure the strain on the surface of FRP bars. This paper conducted compression experiments on the same batch of 25 mm diameter GFRP longitudinal reinforcement used in the RC structure of a bridge in China. It can provide a parameter reference in order to verify that GFRP bars still have certain strength under compressive conditions when applied to large-scale structures. This study uses the technology of digital image correlation (DIC) to measure the surface strain of GFRP. Moreover, the dispersion of compressive strength and compressive elastic modulus of GFRP bars are analyzed, as well. In order to verify that GFRP bars still have certain strength under compressive conditions, this paper provides a parameter reference for large-scale applications.

## 2. Theoretical Basis

### 2.1. DIC Technology

**Figure 1:**
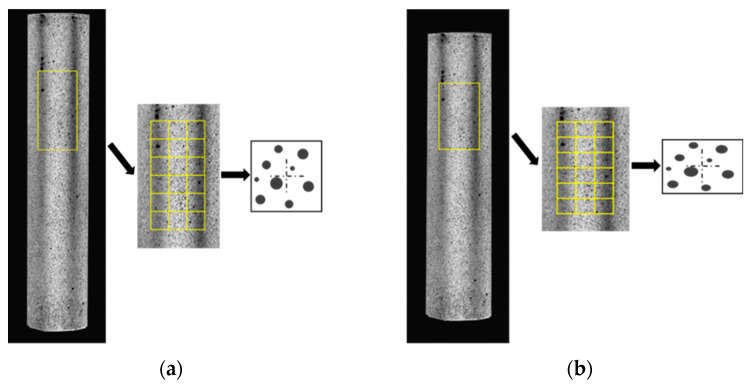
Theory of DIC technology. (**a**) Reference images and reference subsets. (**b**) Target image and target subset.

DIC is a non-contact modern experimental optical measurement technology. Based on the correlation principle between images, it can measure the three-dimensional coordinates, displacement and strain of an object’s surface in the process of deformation by tracking the speckle image on the object’s surface [27]. The basic principle involves comparing an undeformed reference image with a series of deformed images, and dividing the reference image into multiple small squares, called subsets, as shown in Figure 1.

In order to obtain accurate measurement results, speckles of different shapes and sizes are usually created on the test sample by means of a laser, spray paint and natural texture. This gives each subset a recognizable characteristic pattern, and the software is used to search for the subset pattern that is most similar to the reference image in a specific region of the deformed image. The difference between the target subset and the reference subset is the measured displacement. After obtaining the displacement data, the strain can be calculated by dividing the distance change between the spots in the subset (ΔL = Lt − L0) by the original distance between them (L0).

At present, DIC has become a convenient and efficient technical method that is widely used in mechanical experiments to analyze the full-field displacement and strain on the surface of structures that may exist in extreme temperatures or have special sizes, complex structures and high strain rates [28]. The surface of the GFRP bar specimen used in this experiment is rough, and has shallow ribs wound in a clockwise direction. Grinding and pasting the strain gauge will have a great impact on the strength. In addition, due to the anisotropy of GFRP and eccentric compression during loading, the strains at different positions on the surface of the specimen are different during compression, and the failure position is variable [29]. Therefore, the use of DIC equipment to observe strains has obvious advantages for both the operation and accuracy of results. 

### 2.2. Probability Distribution Theory

For the probability distribution of mechanical properties of materials, the commonly used functions mainly include normal distribution, lognormal distribution, gamma distribution, Weibull distribution, etc. Among them, the traditional RC structure is mostly fitted via normal distribution, and its application to composite materials is mostly based on empirical assumptions. Studies have shown that Weibull distribution [30] is more suitable for analyzing the probability distribution of FRP material strength. From the perspective of probability theory and statistics, all continuous probability distributions can be expressed using the probability density function (PDF) and the cumulative probability density function (CDF). The following four commonly used models are introduced:

(i) Normal Distribution

Normal distribution is also known as Gaussian distribution. If random variable *x* follows a normal distribution with mathematical expectation μ and variance σ^2^, denoted as N (μ, σ^2^), then its probability density function is:

(1) $$f(x)=\frac{1}{\sqrt{2\pi \sigma }}\exp\left(-\frac{1}{2}{\left(\frac{x-\mu }{\sigma }\right)}^{2}\right)$$

The probability density function is normally distributed. The expected value μ determines the position, and the standard deviation σ determines the amplitude of the distribution. The normal distribution for μ = 0 and σ = 1 is the standard normal distribution.

(ii) Lognormal Distribution

Lognormal distribution means that the logarithm of a random variable follows normal distribution. In other words, the random variable follows lognormal distribution. Lognormal distribution is very close to normal distribution, and its probability density function is:

(2) $$f(x)=\frac{1}{\sqrt{2\pi x\sigma }}\exp\left(-\frac{1}{2}{\left(\frac{\ln\left(x\right)-\mu }{\sigma }\right)}^{2}\right)$$

(iii) Gamma Distribution

Gamma distribution is a continuous probability function in statistics, and is a very important distribution in probability statistics. Its probability density function is:

(3) $$f(x)=\frac{\alpha }{\beta^{\alpha }\Gamma \left(\alpha \right)}x^{\alpha -1}\exp\left(\frac{x}{\beta }\right)$$

where α > 0 is the shape parameter, and β > 0 is the inverse scale parameter.

(iv) Weibull Distribution

Weibull distribution is widely used in the field of civil engineering materials. In 1951, Swedish mathematician Waloddi Weibull [31] explained the definition of this distribution in detail based on the weakest chain theory, which was subsequently used in the strength analysis of materials. Weibull distribution has many forms, among which the most widely used is two-parameter Weibull distribution, which is determined by two parameters: shape and scale.

(4) $$f\left(x;\lambda ;k\right)=\left\{\begin{array}{ll} \frac{k}{\lambda }{\left(\frac{x}{\lambda }\right)}^{k-1}\exp\left[-{\left(\frac{x}{\lambda }\right)}^{k}\right] & x\geq 0\\ \;0 & x<0 \end{array}\right.$$

where *x* is a random variable subject to Weibull distribution, *λ* > 0 is the scale parameter and *k* > 0 is the shape parameter.

According to the properties of probability density function, the cumulative probability density function of Weibull distribution is obtained by integrating Equation (1):

(5) $$\begin{array}{ll} F\left(x;\lambda ;k\right) & =\int_{0}^{x}f\left(x\right)dx\\ & =1-\exp\left[-{\left(\frac{x}{\lambda }\right)}^{k}\right] \end{array}$$

Weibull distribution is correlated with many other distributions, and is determined by two parameters *λ* and *k*. The shape parameter *k* determines the shape characteristics of the curve. When the scale parameter remains unchanged, the PDF function is exponentially distributed when *k* = 1. When *k* = 2, it is Rayleigh distribution. In addition, according to relevant studies, shape parameter k is also used as one of the important indicators to evaluate the mechanical properties of materials. The larger the fitted value of *k*, the better the strength uniformity of materials and the higher the reliability [32].

## 3. Compressive Performance Experiment

### 3.1. Materials

GFRP bars with a diameter of 25 mm provided by Haichuan New Material Technology Co., Ltd. of Shenzhen, China, were selected for the test. The fiber of the GFRP bars used in the experiment was electric glass (E-glass) and the resin in the core material was general standard vinyl ester resin (SWANCOR901). The mechanical properties of the constituent materials (fibers and matrix) are shown in Table 1, Table 2 and Table 3.

**Figure 2:**
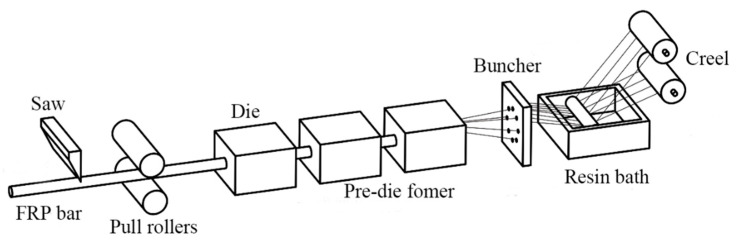
Pultrusion process of GFRP.

GFRP is a composite material prepared from continuous glass fiber and resin via the pultrusion molding method. The related equipment consisted of a fiber creel, a resin bath, a buncher, a mold preformer, pull rollers and a saw. The specific process was divided into the following parts: fiber yarn arrangement → resin impregnation → collection → preforming device → heating and pressure curing device → traction device → cutting device, as shown in Figure 2.

**Figure 3:**
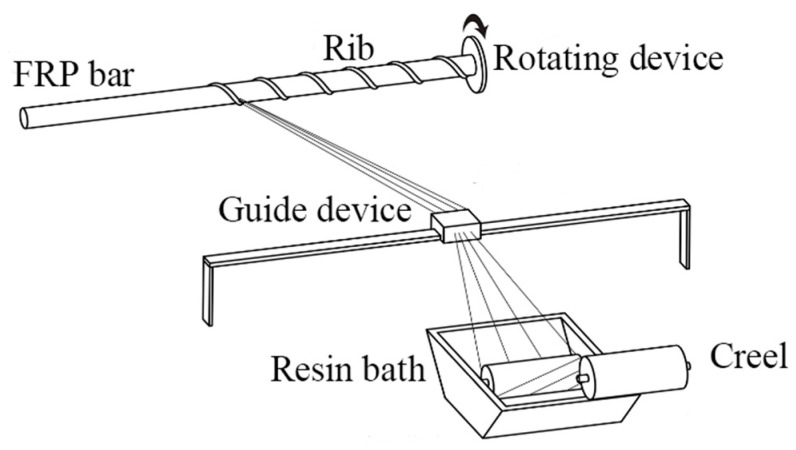
Winding rib process of GFRP.

After the pultrusion process to produce GFRP bars with mainly axial fibers, continuous fiber bundles were impregnated with resin; then, they were wound onto the surface of the bars in a set winding direction and at a set angle. In this way, we could produce GFRP ribs with a specific pitch and angle. Finally, the desired ribbed GFRP rods were obtained using a pressure curing device and a cutting device. The production process is shown in Figure 3.

### 3.2. Experiment Arrangement

The test referred to the “Test Method for Compression Performance of Fiber Reinforced Plastics” (GBT1448-2005) [33], and a MTS-1000 electric servo universal testing machine with a compression capacity of 1000 KN was used to test the compression performance of the GFRP bars.

**Figure 4:**
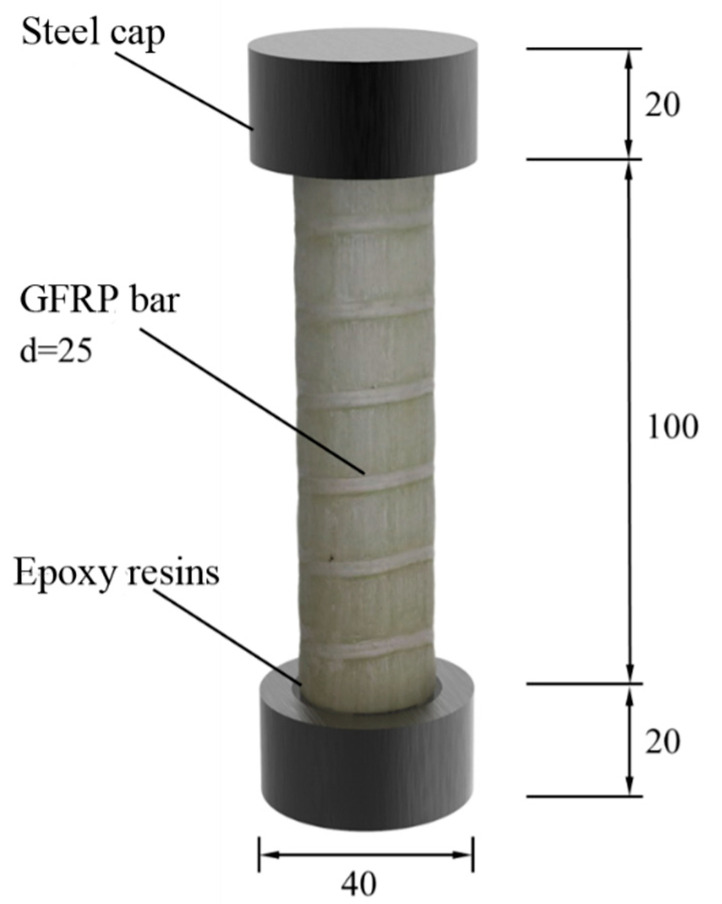
Schematic diagram of specimen size (mm).

**Figure 5:**
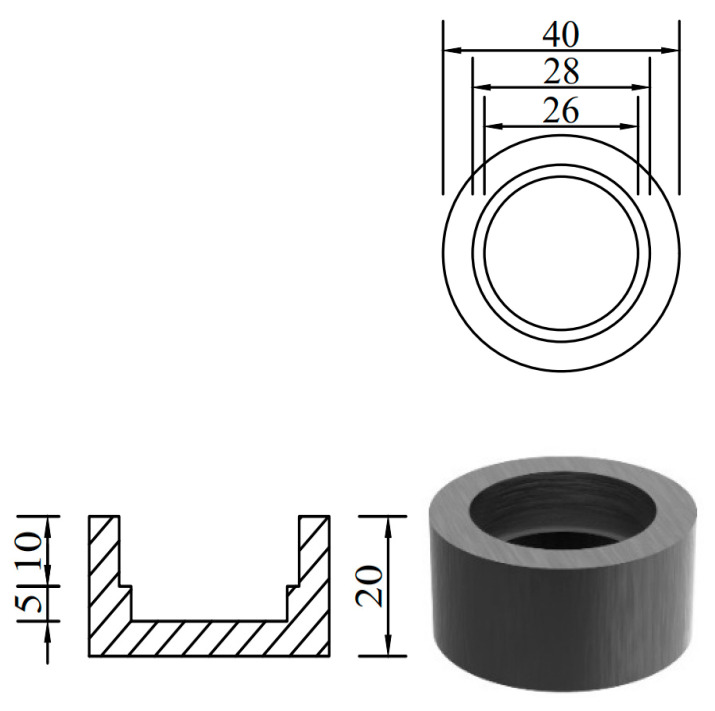
Schematic diagram of steel cap size (dimensions in mm).

In order to prevent stress concentration at the end of the loading process from affecting the testing effect [34], steel caps were used to constrain both ends of the specimen, and epoxy resin was poured into the spare part to fix it. The effective length of the specimen was 100 mm, and the overall size is shown in Figure 4. The size of the steel cap used at both ends of the specimen is shown in Figure 5.

**Figure 6:**
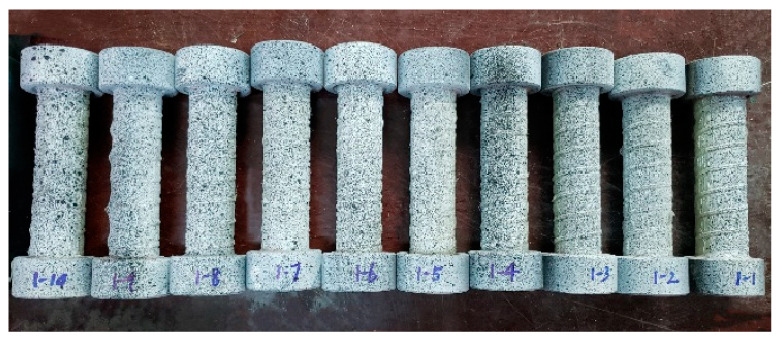
Specimens sprayed with speckles: C1–C10.

The study used DIC to monitor the displacement and strain of specimens during loading. Since DIC is for the image of certain feature points and the random spots sprayed on the specimen surface as the information carrier, the deformation or displacement test system has certain identification requirements for the distribution of feature points on the specimen surface. Therefore, the surface of the test sample should normally be treated with manually generated random speckles according to these conditions prior to the tests. Speckles are made by spraying black and white paint. In this experiment, white paint was selected as the background color and black paint was sprayed randomly to form feature points with an obvious grey level. The paint was sprayed 20 cm away from the test specimens to ensure a uniform distribution and size of the scattered spots. A total of 10 specimens were treated in the experiment, denoted as C1–C10, respectively. The specimens sprayed with speckles are shown in Figure 6.

**Figure 7:**
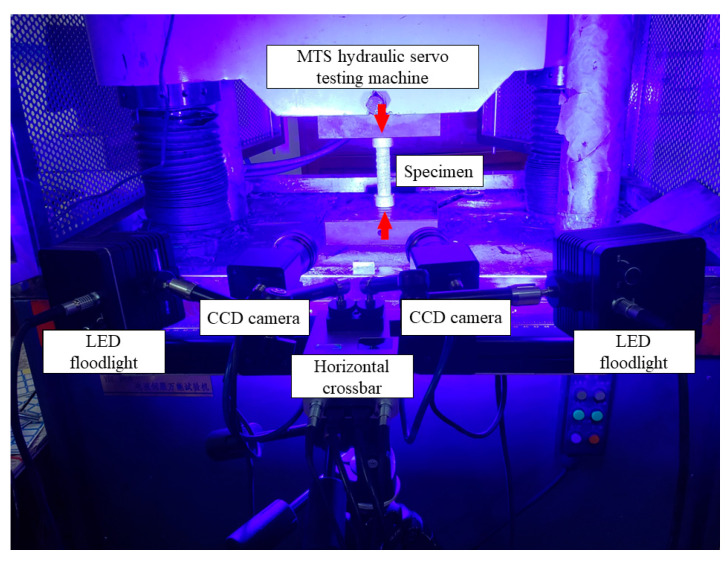
DIC equipment was used to measure the compressive properties of GFRP bars.

The DIC equipment and software used in this experiment were provided by Xintuo 3D Technology Co., Ltd. of Beijing, China. Two charge-coupled device (CCD) cameras were set to collect speckle images on the surface of the tested parts, and the cameras were placed symmetrically at the same angle, ranging from 15 to 30 degrees, with the specimen. The cameras were located on the left and right sides of the horizontal bar on the tripod. We adjusted the three handles on the tripod to ensure that the camera lens was at the same level as the spacing area of the test piece. The light source used two high-power LED lights, whose light intensity and convergence degree could be adjusted according to the needs of the experiment, DIC equipment is shown in Figure 7.

Before the experiment, the CCD camera needed to be calibrated; a calibration plate was placed in the position of the original specimen, and the internal and external parameters of the camera were automatically calculated using the three-dimensional coordinates of the known lattice on the calibration plate and the corresponding image coordinates [35]. The distance between the camera and the sample, the angle between the two cameras and the position of the illumination source in the 3D DIC system were adjusted, and the camera focus was adjusted to make sure the sample image was clear. The experiment began after the calibration of the system parameters. The system analyzed and compared the speckle images at different times using post-processing software, and was able to obtain the out-of-plane displacement of the specimen surface, that is, the spatial displacement of the specimen surface.

The specimens were preloaded to the fixture of the testing machine to press the specimens, and then, the data file storage path was set at the system end. DIC software collection was started on the computer, and then, the compression experiment was started. Table 4 shows the compression test parameters.

## 4. Results

### 4.1. Failure Mode

**Figure 8:**
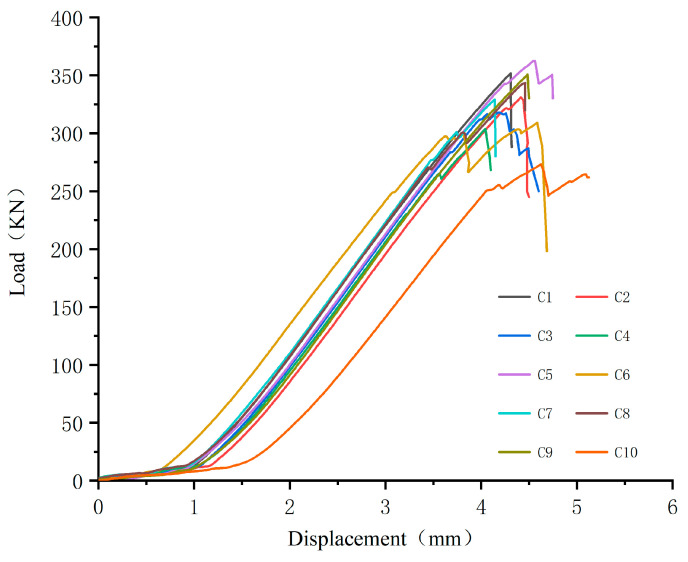
Load–displacement curve.

At the beginning of the test loading when the sample is compressed, a slight sound is heard. With the gradual increase in the bearing capacity of the sample, when the compressive stress in the sample is too large, a resin and fiber peeling phenomenon occurs in the place where the bonding between the glass fiber and the matrix resin is relatively weak. With the continuous increase in the load, the peeling area of the fiber and resin continues to increase until it fails. The failure is accompanied by a loud sound, and the load drops sharply, the test load–displacement curve is shown in the Figure 8.

All the failure modes observed in this paper are very similar. The failure modes of specimens C1–C5 and C6–C10 are shown in Table 5 and Table 6, respectively. Specimens C1–C10 undergo brittle failure caused by the crushing of fibers. Their form is linear elasticity, and there is no yielding or any ductile behavior before failure. In order to observe the failure mode, no speckles were sprayed on the backs of any of the test specimens. As shown in Table 5 and Table 6, all the tested specimens failed due to longitudinal splitting along the fiber direction, and the failure points were mostly located on the middle and lower parts, with obvious fiber peeling. The surface damage of some specimens is not obvious, but several longitudinal cracks along the fiber direction can still be observed after magnification. Specimens with splitting failure at the bottom are more likely to fail under pressure and shear due to the expansion of the GFRP bars during loading, while the deformation at the bottom is limited by the steel cap. Longitudinal splitting failure occurs in specimens are due to the uneven stress on the fibers during the loading process, which leads to failure of the specimen by crushing one side first, and the measured compressive strength is relatively low.

### 4.2. Strain Change (ε)

**Figure 9:**
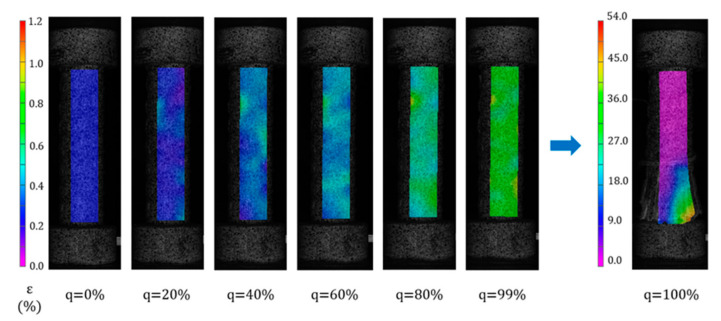
Strain nephogram of specimen C8 under different load levels.

According to the DIC equipment and software analysis, the displacement and strain of the GFRP bars at each point and overall during compression were obtained. For example, a strain nephogram of specimen C8 at different loading stages is shown in Figure 9. The results of the other test specimens are similar to those of C8. It can be seen from the figure that the strain of each part of specimens C1–C10 increases uniformly during the loading process, and there is no stress concentration at the end, which means that the applied load is evenly distributed to the cross-section of the bars. Hence, stress concentration during compression loading can be effectively avoided by adding steel caps at both ends of the GFRP compression specimens. At the failure stage, high strain occurs locally, which is caused by brittle splitting failure of the GFRP bars.

**Figure 10:**
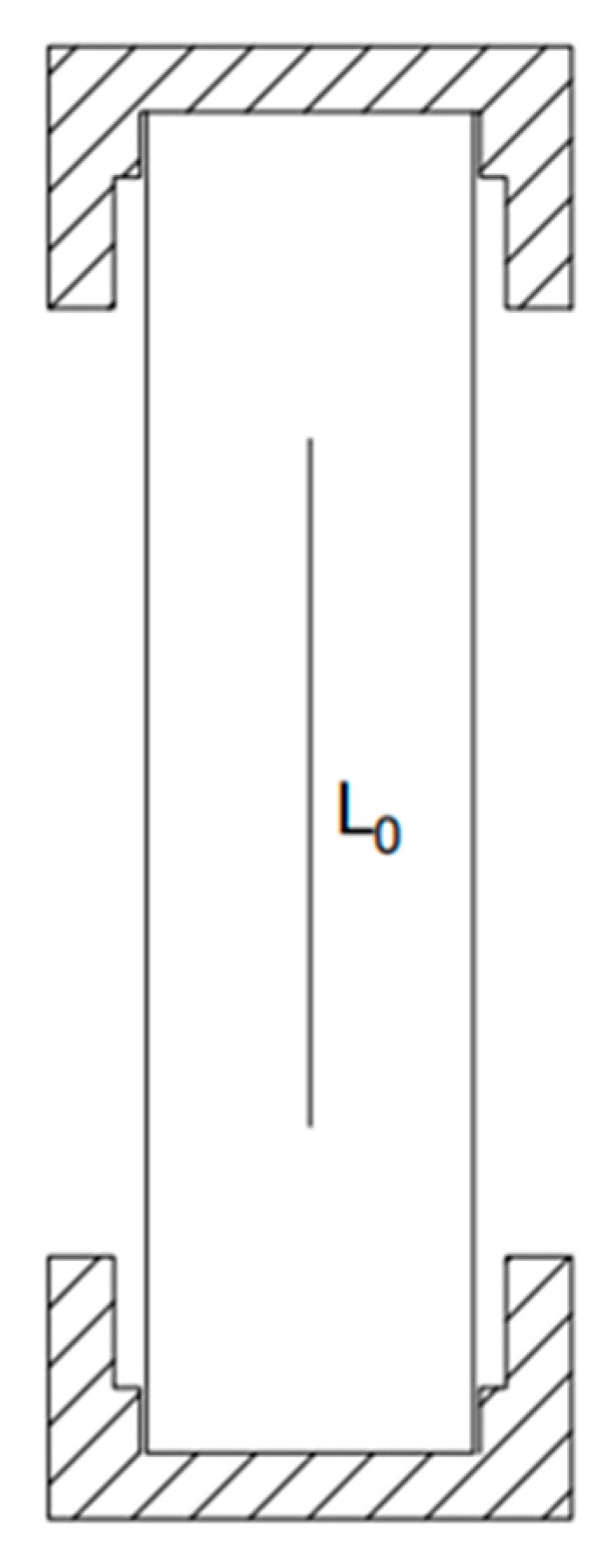
Location diagram of test section L0.

**Figure 11:**
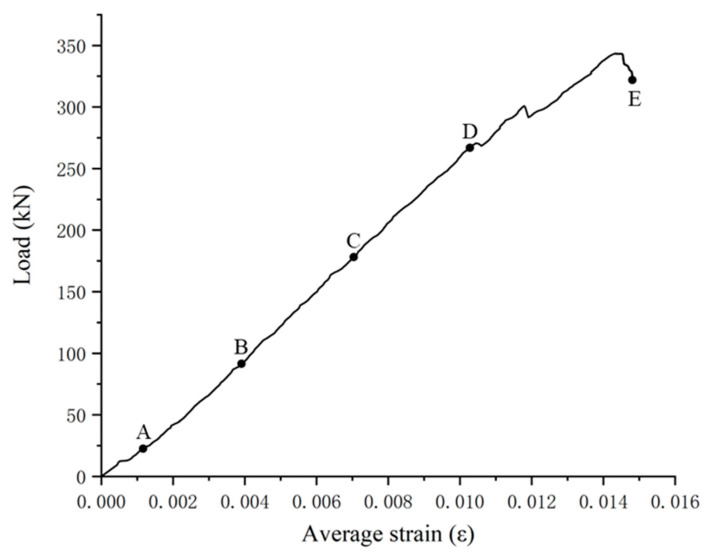
L_0_ load average strain curve of test piece C8 test section (A–E represent different load levels 20% to 100%).

In the DIC software, we selected two points located on the centerline of the test piece with a distance of L_0_ = 80mm, calculated the average strain of the test piece using its ordinate, and obtain the average strain load curve of a single test piece. We corresponded each point on the curve to the strain nephogram at that time, as shown in Figure 10 and Figure 11.

### 4.3. Compressive Modulus of Elasticity

In order to determine the elastic modulus of GFRP bars in compression, it is necessary to calculate the stress. Since the compression test conducted is purely axial (ignoring the small moment due to eccentricity, and measuring the strain at the average position), the calculated stress is the load divided by the cross-sectional area of the reinforcement:

(6) $$\sigma_{C}=\frac{P}{F}$$

(7) $$F=\frac{\pi }{4}D^{2}$$

where: *σ_C_* is the compression stress, unit: MPa; *P* is the maximum load of the test piece, in KN; and *F* is the compression cross section area of the test piece, in mm^2^.

The compression elastic modulus refers to the method in Test Method for Compressive Properties of Fiber Reinforced Plastics (GBT1448-2005), and is calculated according to Formula (3):

(8) $$E_{C}=\frac{\sigma^{\prime \prime }-\sigma^{\prime }}{\varepsilon^{\prime \prime }-\varepsilon^{\prime }}$$

where *E_C_* is the modulus of elasticity of compression, unit: GPa; *σ″* is the compression stress measured when *ε″* = 0.0025, in MPa; and *σ′* is the compression stress measured when *ε′* = 0.0005, in MPa.

**Figure 12:**
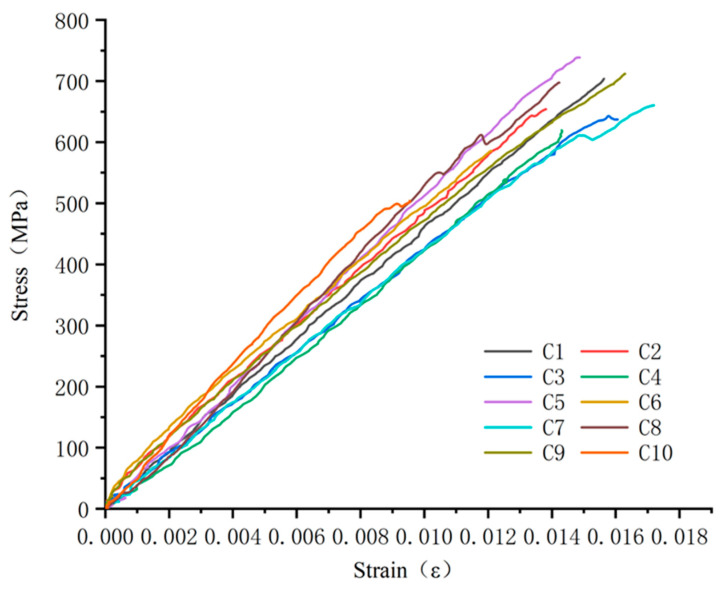
Compression stress–strain curves.

**Figure 13:**
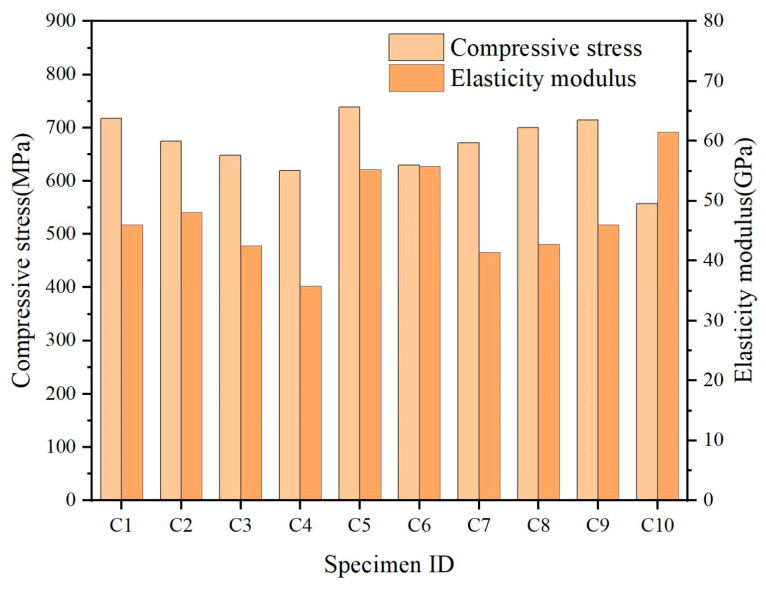
Comparison of compression test results of GFRP bars.

A stress–strain curve of 10 groups of test specimens is shown in Figure 12. It can be seen that the stress–strain of GFRP bars in the compression process is approximately a straight line, and the curves of each specimen gradually separate with the increase in strain and are always at the elastic stage until failure. Brittle failure occurs in all the test specimens. The calculated compressive strength and elastic modulus of 10 groups of test specimens are shown in Figure 13. In order to better observe the dispersion of the experimental results, the average value and standard deviation of each group of data were calculated and are shown in Table 7. This dispersion might be due to manual errors occurring during GFRP production and specimen fabrication, or because the tested specimens might be from different batches of specimens.

## 5. Discreteness Analysis

The main factors affecting the compressive performance of GFRP bars are the materiality of the glass fiber and resin and the manufacturing process. As a typical brittle material, GFRP has a large dispersion of strength and elastic modulus due to the combined effect of initial defects during manufacturing and the external environment in practical engineering. The uncertainty of the strength of this material can be analyzed using the method of probability statistics. Due to the large diameter of the 25 mm GFRP bars studied in this paper, it was difficult to find data for studies conducted on the same dimensions. According to the research results in the literature [21], it was found that the differences in the compressive strength and dispersion of GFRP bars of different diameters were small. Therefore, we compared the discrete differences in the experimental results of the compressive strength and compressive elastic modulus of GFRP bars under different size conditions. The arithmetic mean was then taken for the dispersion of different diameters, as shown in Table 8.

It can be seen from the table that the dispersion of the compressive strength of GFRP bars is generally large. In this paper, the elastic modulus obtained via DIC strain measurement calculation is larger than that obtained using other methods. The most commonly used distribution function was selected as the probability distribution model to analyze the compressive strength and elastic modulus of GFRP bars, and the fitting results were compared to obtain the optimal solution.

### 5.1. Compressive Strength

**Figure 14:**
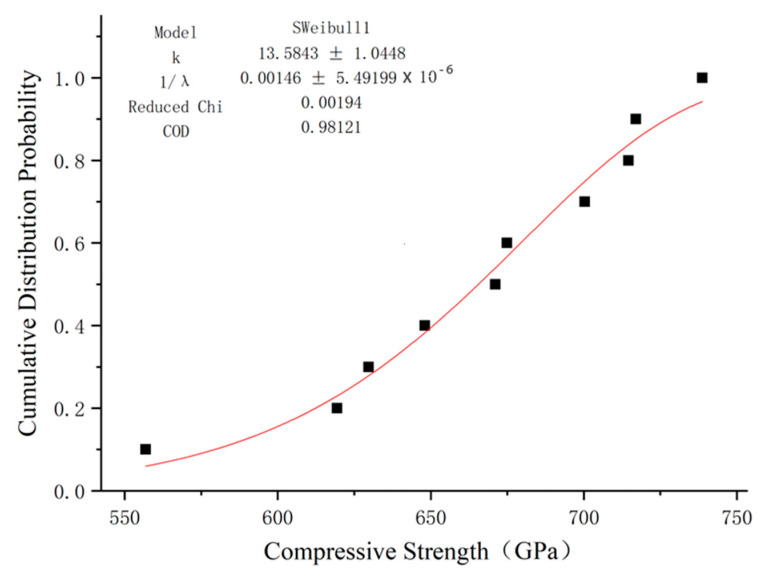
Fitting of compressive strength function of GFRP bars via Weibull distribution.

**Figure 15:**
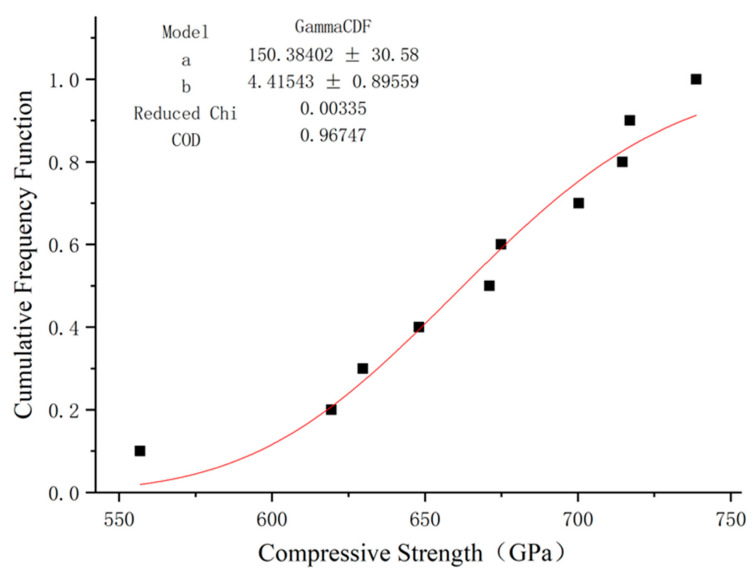
Fitting of compressive strength function of GFRP bars via gamma distribution.

There are limited studies on the use of distribution functions to describe the compressive strength and elastic modulus of GFRP. In addition to Weibull distribution, gamma distribution is often used in engineering to analyze the mechanical properties of fiber composites [37]. In this paper, these two distributions were used to fit the compression elastic modulus results of GFRP bars, and the fitting results are shown in Figure 14 and Figure 15.

We used the Weibull distribution fitting results to obtain a shape parameter k of 13.58 and a size parameter λ of 684.93. The distribution equation of compressive strength of GFRP bars is:

(9) $$F(x)=1-\exp\left[-{\left(\frac{x}{684.93}\right)}^{13.58}\right]$$

By observing the cumulative probability distribution image of the GFRP bars’ compression strength using Weibull distribution, it can be seen that this distribution can fit the test results well, and its determination coefficient R2 is close to 1. gamma distribution can also be fitted, but the effect is quite poor, so Weibull distribution is the most appropriate method of fitting the compressive strength of GFRP bars.

### 5.2. Compressive Modulus of Elasticity

For the results of the compressive elastic modulus of GFRP bars, the fitted Weibull function shape parameter *k* is 6.55, the size parameter *λ* is 48.40 and the distribution equation of compressive strength of GFRP bars is:

(10) $$F(x)=1-\exp\left[-{\left(\frac{x}{48.4}\right)}^{6.55}\right]$$

**Figure 16:**
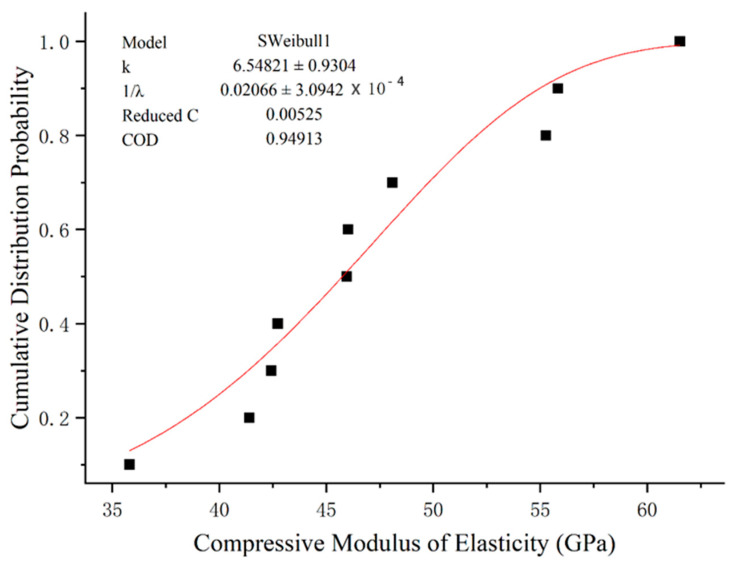
Fitting of compression elastic modulus function of GFRP bars via Weibull Distribution.

**Figure 17:**
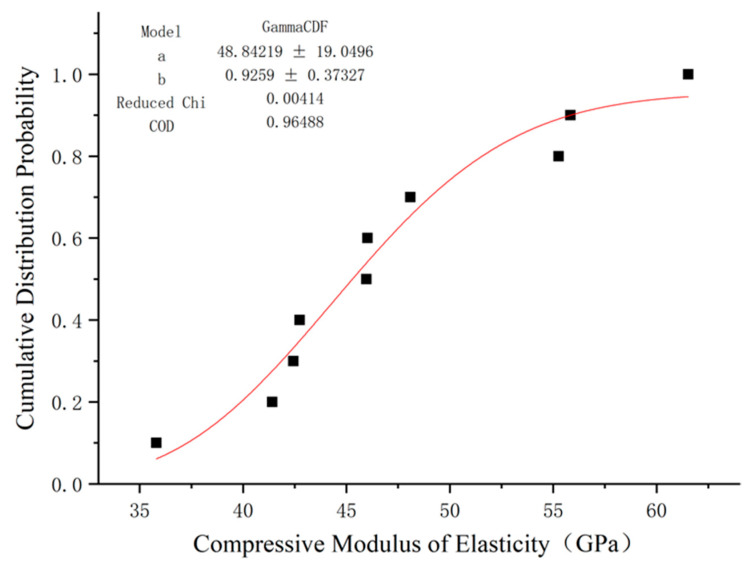
Fitting of compression elastic modulus function of GFRP bars via gamma distribution.

Similarly, a fitting curve that obeys gamma distribution can be obtained. By comparing the fitting results, it can be seen that the determination coefficient R2 of gamma distribution fitting is closer to 1 than Weibull distribution fitting, and the fitting effect is better. Therefore, it is better to use the gamma function when determining the compressive elastic modulus of GFRP bars and the fitting results are shown in Figure 16 and Figure 17.

## 6. Conclusions

The DIC technique was used to analyze the strain evolution of GFRP bars with a 25 mm diameter during compression. Moreover, Weibull distribution and gamma distribution were used to fit the compressive strength and compressive elastic modulus of GFRP bars as there are limited studies on the use of distribution functions to describe the compressive strength and elastic modulus of GFRP. The experiment provides a parameter reference for the application of large-size GFRP bars used in bridge pile foundation projects, and the main conclusions of the experiment are as follows:

(i) DIC technology successfully determined that the surface strain of GFRP bars is uniformly distributed and approximately linearly increased, and the brittle splitting failure of GFRP bars happened due to locally occurring high strain at the failure stage.
(ii) All the tested specimens failed due to longitudinal splitting along the fiber direction, and the failure points were mostly located at the middle and lower parts, with obvious fiber peeling.
(iii) Stress concentration during compression loading can be effectively avoided by adding steel caps at both ends of the GFRP compression specimens.
(iv) The probability distribution of the compressive mechanical properties of GFRP bars was verified through experiments. The average compressive strength of GFRP bars is 667.05 MPa and follows Weibull distribution. Moreover, the average compressive elastic modulus of GFRP bars is 47.51 GPa and follows gamma distribution.

## Figures and Tables

**Table 1:** Properties of glass fiber.

Fiber Type	Monofilament Diameter (μm)	Density (g/cm^3^)
E-glass	14 μm	2.51

**Table 2:** Properties of matrix.

Matrix Type	Tensile Strength (MPa)	Tensile Modulus (GPa)	Density (g/cm^3^)
Vinyl ester resin	92.5	3.4	10

**Table 3:** Properties of GFRP bars.

Diameter (mm)	Tensile Strength (MPa)	Tensile Modulus (GPa)	Fiber Content (%)	Rib Spacing (mm)
25	747.4	52.3	78	10

**Table 4:** Compression test parameters.

Number of Specimens	Free Segment Length (mm)	Sampling Frequency (Hz)	Loading Rate (mm/min)
10	100	2	2

**Table 5:** Failure mode of specimens C1–C5.

Num	C1	C2	C3	C4	C5
Image	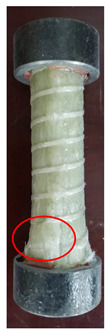	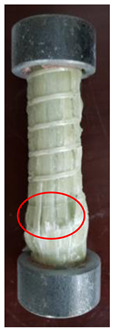	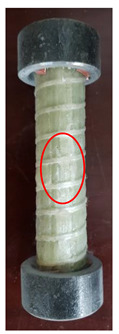	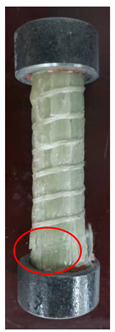	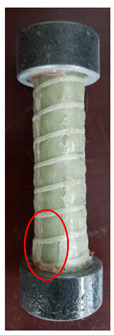
Details	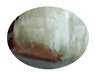	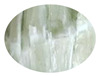	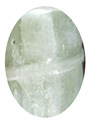	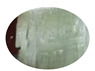	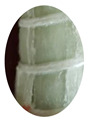
Failure form	Obvious splitting failure at the bottom	Obvious splitting failure at the bottom	Longitudinal splitting failure	Obvious splitting failure at the bottom	Overall longitudinal splitting failure

**Table 6:** Failure mode of specimens C6–C10.

Num	C6	C7	C8	C9	C10
Image	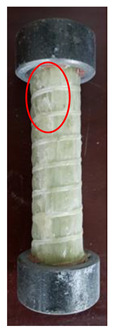	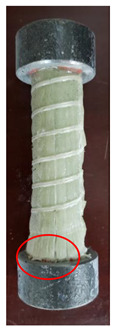	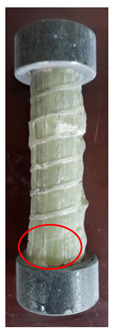	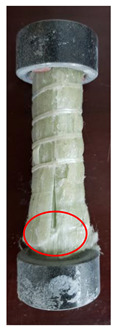	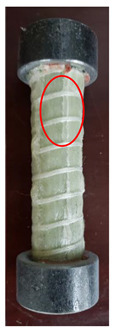
Details	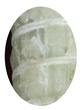	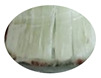	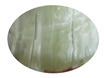	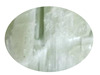	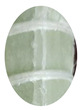
Failure form	Longitudinal splitting failure	Obvious splitting failure at the bottom	Obvious splitting failure at the bottom	Obvious splitting failure at the bottom	Longitudinal splitting failure

**Table 7:** Compression test results of FRP bars.

Num	Compressive Strength (MPa)	Compression Modulus (GPa)	Peak Strain
C1	717.02	45.96	0.016
C2	674.85	48.09	0.014
C3	648.01	42.43	0.016
C4	619.37	35.80	0.014
C5	738.69	55.27	0.015
C6	629.68	55.83	0.012
C7	671.14	41.41	0.017
C8	700.27	42.74	0.016
C9	714.61	46.02	0.016
C10	556.82	61.53	0.009
μ	667.046	47.508	0.0145
σ	52.207	7.442	0.00229
COV.	0.08	0.16	0.16

**Table 8:** Comparison of experimental dispersions of GFRP bars.

	Diameters (mm)	Compressive Strength (MPa)		Compression Modulus (GPa)	
		σ	COV	σ	COV
This study	25	52.21	0.08	7.44	0.16
Yu et al. [21]	8,10,12	113.00	0.16	3.32	0.06
Liu et al. [22]	16	45.78	0.10	1.6	0.035
AlAjarmeh et al. [36]	9.5,15.9,19.1	81.9	0.09	1.48	0.03

## Data Availability

Not applicable.
